# The National Medical Union (Official)

**Published:** 1914-02-28

**Authors:** 


### THE NATIONAL MEDICAL UNION (Official).

Provisional Federating Council.

There will be a meeting of the Provisional Federating Council at the offices of the Union, 346 Strand, on Saturday, March 21, at 4.30 p.m., to consider mainly the subject of policy. Members are invited to forward any resolutions to the hon. secretaries which they may wish to be brought before the Council, not later than Saturday, March 7, in order that these may be issued on the agenda for the meeting, which will be sent out during the subsequent week.

Robert Carswell,
E. Arthur Dorrell,
Hon. Secretaries.

Manchester Executive Committee.

The Executive Committee of the National Medical Union, Manchester District, held a meeting on Tuesday, February 17. The following gentlemen attended: Mr. W. Coates, C.B. (in the chair), Mr. G. A. Wright, Drs. Pinder, Stenhouse, Lowe, MacMillan, Grange, A. G. Clarke, Malim, and Prowse. Dr. F. Heatherley, hon. secretary of the Liverpool Non-Panel Union, was present as a visitor.

Drs. Lowe and Prowse reported attendance at the Federating Council meeting of January 31, and gave some account of the proceedings of that body. A resolution was adopted calling attention to the importance of taking active measures to enrol all non-panel men as members, and of issuing at an early date a clear statement of the amendments to the Insurance Acts which are considered necessary.

A sub-committee was appointed to draw up a statement of the chief evils resulting directly from the present administration of the medical benefits under the Acts. This will come up for consideration at the next meeting of the committee, with a view to its circulation to all members and its publication in the Press.

Unallotted Medical Benefit.

On February 16 a report appeared in the *Times* of an interview between a deputation of panel doctors and Mr. Masterman, the late member for Bethnal Green, who, it will be remembered, was making his unsuccessful bid for re-election to that constituency.

The deputation wanted to know if the unallotted funds under the Insurance Act would be paid to the panel doctors. To this question Mr. Masterman was said to have given an answer in the affirmative, and the assurance that even if counsel's opinion should once more prove unfavourable the panel doctors are to get the money.

This reply is all the more extraordinary, even making allowance for its having been given in the course of an election, when the previous history of this question and of the legal restrictions laid down in the Act as to the method by which panel doctors are to be remunerated are taken into consideration. These funds, which in the case of the London area must by this time amount to some £200,000, have mainly accumulated, it is well to recall, through the determination of some 400,000 insured persons in London to employ the doctor they prefer rather than one from the limited panel list of the county, and to do so at their own expense. The accumulated surplus represents in effect so many insurance premiums, contributed under compulsion in accordance with Mr. Lloyd George's oft-repeated and unfulfilled promise to the worker of "the doctor of his choice." It is, therefore, difficult to see how the funds could in equity be given as a bonus to panel doctors.

But in any case they are not in the gift of Mr. Masterman, since the National Insurance Act of 1911 provides that the Insurance Committees, and not candidates for Parliamentary elections, shall be the proper authorities to administer medical benefit. The manner of that administration, in so far as it affects the payment of panel doctors, is further provided for in more than one subsequent regulation of the Insurance Commissioners, and in the contracts between the two parties, under which remuneration is to be calculated on the basis of the mean average number of patients on their lists on the first and last day of each quarter. The London Insurance Committee on more than one occasion, notwithstanding its chairman and the ingenious sophistry of the Chancellor of the Exchequer, refused to be pushed into dividing this large sum among panel doctors, but took counsel's opinion.

An opinion given by Mr. Danckwerts, K.C., last September was entirely against any such distribution, and need only be quoted in part. He said: "I can find no warrant in the Act or elsewhere in any regulation of the Insurance Commissioners which has been placed before me for the Parliamentary statement that the whole fund for medical benefit is divisible among the practitioners on the panel. To my mind it is not so. Under Section 54 (1) the Health Insurance Fund can only be resorted to by the Insurance Committee to meet expenditure properly incurred by the committee; there is nothing in the Act to affect this root principle. . . . Now the practitioners on the panel act and have taken service under a written contract (Form M.B. 4), and under clause 10, and Schedule IV., thereof they are entitled to be paid definite sums calculated at so much per head for the insured persons on their list. I cannot find any right in the contract to claim more. It follows necessarily that the Committee cannot lawfully or properly pay more out of public funds; the Committee is not entitled to make presents or distribute largesse among the panel doctors."

If this opinion is reliable Mr. Masterman might with advantage study the concluding sentence. For if the London Insurance Committee, as the legally appointed authority to administer medical benefit, is not entitled to make presents or distribute largesse, it seems as though Mr. Masterman's promise to his panel constituents will prove to be a very dry bone indeed, and especially since he has failed to be re-elected to Parliament.

But if his statement is to be taken as an announcement by the Government of an intention to legalise, by means of an amending Act, such disposal of a sum of money amounting in all to more than a million pounds, then it is to be hoped that the proposal will be strenuously opposed by those to whom the money rightly belongs.

### The Profession of Medicine.

### A JURY'S JUSTIFIABLE CENSURE.

If cases similar to that unfolded at an inquest in Wandsworth a week ago are at all common, it is not to be wondered at that juries and the rest of the public form a bad opinion of what they know as "medical etiquette." The popular conception of this is a rigid and binding code of laws, overriding all common sense and humanity; whereas in truth it is merely a rule of conduct based upon the interests of the public and the ordinary standards of gentlemanly behaviour. But it is not astonishing that it should be misunderstood when made a cloak and an excuse for conduct such as that of the medical man whom the Coroner and his jury very properly censured at Wandsworth.

### Pique and Pettiness.

The facts of the case, as reported in the press, are briefly these. An insured youth fell ill, and his father summoned his panel doctor, Mr. John Ffrench Blake. This practitioner said there was nothing the matter; but seems to have changed his opinion the next morning, when again summoned, for he then diagnosed cerebral irritation. In the afternoon the boy was worse, and the paneller was once more summoned: he promised to come at 5 p.m., but the father called in another practitioner because he saw that his son was dying. This was at four o'clock. When Mr. Blake arrived he was told that the other doctor was with the boy at that moment, and thereupon he emphatically refused even to see the patient, on the ground that another doctor had been called in; the boy died at a quarter past five. When Mr. Blake was applied to for a certificate he declined to give it on the ground that it was evening, and said he would give one at a stated time next day.

Questioned about these extraordinary proceedings in the Coroner's court, Mr. Blake sheltered himself behind pretexts of professional ethics. His answers to the Coroner's questions betray, we do not hesitate to say, a most lamentable ignorance both of "medical etiquette" and of common humanity. It is not surprising that his attitude elicited some caustic criticisms from the Coroner, who endorsed the censure which the jury expressed in their verdict.

The details of this regrettable story prove nothing, of course, beyond the fact that there may be men in the medical profession who cannot be trusted to exhibit kindliness and gentlemanliness. Unfortunately, such incidents are often viewed in an entirely wrong light by the public. The impression is gained that the manifest wrongheadedness displayed in such a case as this is not really the fault of the individual, but the result of the mysterious "medical etiquette." The real lesson is exactly the opposite—namely, that the conduct of the panel practitioner in this particular case was as wholly opposed to professional ethics as it was to common sense; that the excuses he offered would not hold water for a moment; and that true "professional etiquette" is wholly salutary and beneficent both for doctors and their patients. It is also true that a man who has the instincts of a gentleman does not need to worry much about this code of behaviour, since he is little likely to transgress it. If the public could grasp this, there would be much less grumbling heard about the stupidity of medical ethics; but it may be admitted that the misapprehensions which are current are to a large degree the fault of those members of the medical profession who from time to time misrepresent its principles, as in the case set forth above.

### Elementary Principles.

There is no rule in medical ethics which is not liable to suspension or abrogation in a case of emergency, especially where that emergency is so grave that life itself is in jeopardy. Nor is there any custom which entitles a doctor to refuse a death certificate when asked for one, unless he is in such doubt about the case that he proposes to notify the Coroner. To postpone writing out such a simple thing as a death certificate merely to suit one's own convenience, while neglecting that of other people, is also a proceeding which no professional code would sanction for a moment. It were greatly to be desired that as much publicity should attend some simple exposition of the true facts of medical ethics and custom as has attended the circumstances of this Wandsworth inquest.

It is perfectly true that when a patient who is being attended by one doctor desires to obtain the opinion of another he must either dismiss the first one or arrange for the two to act in consultation. That is a reasonable proposition, for it is manifestly against the interest of a patient to follow the advice of two separate practitioners, each of whom is in ignorance of the treatment ordered by the other. It is also true that a doctor has good ground for remonstrance if he finds that another one has been consulted behind his back and without his knowledge. But the mere fact that this custom exists in the interests of the patient and not at all in those of the medical profession shows that it is not meant to apply to cases of emergency. In this instance the doctor in attendance was actually summoned, and it was only because he could not come for two hours and because the patient was obviously dying that another practitioner was called in. There was no question of his supersession, and he had no legitimate complaint against the patient's relatives.

